# Wind and route choice affect performance of bees flying above versus within a cluttered obstacle field

**DOI:** 10.1371/journal.pone.0265911

**Published:** 2022-03-24

**Authors:** Nicholas P. Burnett, Marc A. Badger, Stacey A. Combes

**Affiliations:** 1 Department of Neurobiology, Physiology, and Behavior, University of California at Davis, Davis, California, United States of America; 2 Department of Computer and Information Science, University of Pennsylvania, Philadelphia, Pennsylvania, United States of America; RMIT University, AUSTRALIA

## Abstract

Bees flying through natural landscapes frequently encounter physical challenges, such as wind and cluttered vegetation, but the influence of these factors on flight performance remains unknown. We analyzed 548 videos of wild-caught honeybees (*Apis mellifera*) flying through an enclosure containing a field of vertical obstacles that bees could choose to fly within (through open corridors, without maneuvering) or above. We varied obstacle field height and wind condition (still, headwinds or tailwinds), and examined how these factors affected bees’ flight altitude, ground speed, and side-to-side casting motions (lateral excursions). When obstacle fields were short, bees flew at altitudes near the midpoint between the tunnel floor and ceiling. When obstacle fields approached or exceeded this midpoint, bees tended to increase their altitude, but they did not always avoid flying through obstacles, despite having the freedom to do so. Bees that flew above the obstacles exhibited 40% faster ground speeds and 36% larger lateral excursions than bees that flew within the obstacle fields. Wind did not affect flight altitude, but bees flew 12–19% faster in tailwinds, and their lateral excursions were 19% larger when flying in headwinds or tailwinds, as compared to still air. Our results show that bees flying through complex environments display flexibility in their route choices (i.e., flying above obstacles in some trials and through them in others), which affects their overall flight performance. Similar choices in natural landscapes could have broad implications for foraging efficiency, pollination, and mortality in wild bees.

## Introduction

Nectivorous insects such as bees (Hymenoptera: Apoidea) provide important ecosystem services by pollinating wild plants and crops, and these services are intimately linked to their ability to successfully move through complex habitats while foraging for floral resources [1]. Bees typically move between their nest site and food sources by flying, sometimes over distances of several kilometers [2], yet many physical features of bees’ habitats can make flight a demanding, dangerous, and energetically expensive task [3–5]. Bees regularly fly through environments containing unpredictable winds and cluttered vegetation, each of which can pose distinct challenges to flight [5–8]. Furthermore, the heterogeneity of natural landscapes results in multiple route options for bees, so the unique behavioral choices made by individual bees can dictate the microhabitat (e.g., wind, clutter) that bees encounter, and thus the specific flight challenges that they must overcome [9]. Although bees foraging in wind and around vegetation is an everyday sight, we know surprisingly little about the effects of these habitat features on the flight behavior and performance of bees.

Cluttered vegetation can pose mechanical challenges to flying bees, but at the same time it provides visual landmarks that help bees navigate their environment [9–12]. The structure of vegetation can vary in many ways, including plant density, plant height, leaf size, and branch size [13–15]. Bees find navigable paths through clutter by using both brightness gradients within gaps (where brightness increases with gap size) [11] and optic flow, which is the apparent motion of the landscape moving past a bee’s eyes [16–19]. In particular, optic flow helps bees navigate clutter under a variety of conditions because it depends on the speed of a bee (relative to an obstacle) as well as the bee’s proximity to the obstacle. Thus, bees can use optic flow to gauge their distance from an obstacle [16, 17], to reduce their flight speed as they approach an obstacle [20], to maintain their flight speed relative to an obstacle despite external wind [21], and to center themselves within a flight corridor, by moving laterally to balance optic flow across their left and right eyes [12, 18, 19]. In addition, the difference in optic flow produced by nearby obstacles versus the background helps bees gauge the dimensions and distance of obstacles [22, 23]. Bees can enhance the visual information they receive from obstacles in the environment by performing side-to-side casting maneuvers as they fly or by slowing down and visually inspecting obstacles before continuing their flights, a behavior often associated with learning the layout of a new environment [22–24]. Overall, visual information is crucial for flying bees, and obstacles (e.g., cluttered vegetation) help provide the signals necessary for bees to successfully transit these structures. While visually guided bee flight is well-studied in simplified, artificial laboratory settings, we know little about how these principles operate in more complex, variable environments.

Cluttered vegetation also poses a flight hazard because collisions with vegetation can lead to irreparable wing damage [4], which impairs flight performance and is associated with higher mortality in bees [4, 7]. To avoid collisions, bees flying through cluttered vegetation (or other obstacles) can use the visual information they gather to execute rapid lateral or vertical maneuvers, perform braking maneuvers, or take more sinuous paths around the obstacles [6, 7, 25]. Much of our empirical knowledge about obstacle traversal by bees is based on experiments in which bees are forced to transit simplified obstacles or apertures. Although these experiments reveal the physical mechanisms by which bees can traverse obstacles, they provide no information about how bees in nature negotiate complex physical environments, particularly given that bees have a variety of route choices available to them in natural settings, the most general of which are flying within (between) obstacles or bypassing obstacles entirely (e.g., flying above them). The choice to fly between obstacles may carry an increased risk of wing or body collisions [4], but it also provides strong visual signals that help bees control their ground speed and flight path (e.g., by centering themselves between lateral obstacles) [26]. Examining bee flight performance in the context of route choice and flight trajectory can help reveal how bees weigh the risk of collisions with obstacles against the enhanced visual information and other potential benefits associated with flying through cluttered vegetation.

Bees flying in natural environments also regularly encounter wind, which can vary in speed, direction, and structure (e.g., periodic vortices, fully mixed turbulence), and each of these attributes of wind can affect flight performance in different ways. Bees flying into a steady wind can modify their flight speed relative to the air, to compensate for wind and maintain a constant speed relative to the ground [21]. However, headwinds containing periodic vortices (such as those shed behind a branch in wind) or fully mixed turbulence destabilize flying bees, impairing their ability to maintain a constant body orientation around the roll axis [5, 27, 28]. To compensate for this reduced roll stability, bees can increase the flapping frequency of their wings, modulate stroke amplitude to produce corrective asymmetries, and/or extend their legs, but many of these responses are likely to increase the energetic cost of flight [5, 27]. Isolated gusts of wind can also cause flight instabilities, which trigger a suite of passive responses in bees such as altered body angle and flight speed, followed by active responses to return to their original body orientation and speed [29–31]. In addition, wind can increase the danger associated with some common flight maneuvers such as landing, causing bees to collide with landing surfaces rather than gradually slowing down to land [29, 32]. Despite the growing body of research in this area, our knowledge about how wind affects flying bees remains limited to a fairly narrow set of experimental conditions, such as flight in open air streams without any physical clutter [5, 27, 31] or flight through vortices generated downstream of a single object in wind [28, 29]. Thus, we know little about how wind affects the behavioral choices and flight performance of bees flying in more natural, cluttered habitats.

Bees encountering a large patch of cluttered vegetation in nature can choose to fly through the clutter or to fly above it, and they often make this choice while also contending with wind blowing in different directions relative to their flight path. To understand how wind, clutter, and route choice affect the flight performance of bees traversing complex environments, we filmed wild-caught honeybees (*Apis mellifera* Linnaeus 1758) flying through a laboratory enclosure containing a field of vertical obstacles. We varied obstacle field height (ranging from 11 to 127 mm tall, within a 191-mm tall enclosure) and wind condition (still air, headwinds or tailwinds). Obstacles were arranged in longitudinal rows, providing two unobstructed flight corridors between the obstacles, which were 57 mm wide (~3X wider than a bee’s average wingspan [33]); thus, bees choosing to fly within the obstacles were not required to perform lateral maneuvers, but they did travel through a narrower corridor than those flying above the obstacles. We chose *A*. *mellifera* as a model organism because it is an important pollinator [34], its flight behavior is well-studied [17, 33], and it shows little variation in body size [35], which helps eliminate one known source of variation in flight performance [6, 7, 36]. We reconstructed bees’ flight paths and used these data to answer two primary questions: (1) Does obstacle field height or wind condition affect bees’ flight altitudes? (i.e., their route choice, flying above vs. within the obstacle field), and (2) Does flight altitude or wind condition affect bees’ flight performance?

## Materials and methods

### Experimental set-up

**Fig 1:**
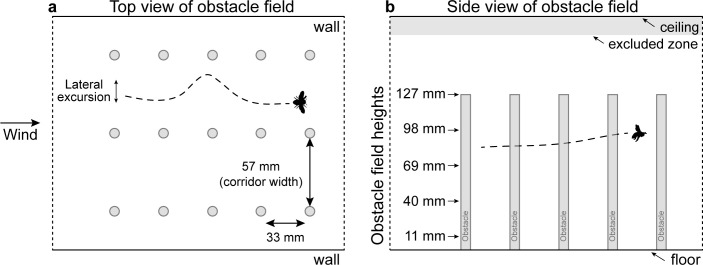
Schematic of experimental obstacle field. (a) Top view: obstacles in each field were arranged in three rows of five vertical columns each, forming two 57-mm wide, unobstructed flight corridors down the longitudinal axis of the enclosure. Bees’ flight paths within the horizontal plane (from above) were used to calculate lateral excursions (interquartile range of lateral positions throughout the flight). (b) Side view: the total height of the tunnel was 191 mm, and five obstacle field heights, ranging from 11 to 127 mm, were tested. Flights that crossed into the ‘excluded zone’ (within 15 mm of the ceiling; gray area on figure) were discarded.

Experiments were conducted in a laboratory flight tunnel (20.0 x 19.1 x 115.0 cm; width x height x length), which had a working section similar in size to tunnels used in other studies of bee flight behavior [18, 25, 26, 37]. There was a field of obstacles (hereafter referred to as the ‘obstacle field’) in the middle of the tunnel, which consisted of vertical columns (diameter = 7 mm) arranged in three parallel rows of five obstacles each, running along the length of the tunnel (Fig 1A). Obstacles were made of dark green, cylindrical blocks (LEGO, Billund, Denmark) that contrasted with the black and white speckled pattern of the tunnel’s walls (NuWallpaper NU2673, Wall Pops, Randolph, MA, USA; S1 File), and flight data indicated that bees were able to detect and avoid these obstacles. All obstacles within an obstacle field were of the same height, and the obstacles extended only partway to the tunnel’s ceiling, allowing bees to fly either within or above the obstacle field. The total height of the tunnel was 191 mm and the obstacle field heights tested were 11, 40, 69, 98, or 127 mm (Fig 1B), so a minimum of 64 mm (~1/3 of the total vertical height) between the top of the obstacle field and the ceiling remained free of obstructions. We consider the 11-mm obstacle field as a control for the presence of obstacles because this obstacle field was too short for bees to fly within. There were approximately 20 mm between the outer rows of the obstacle field and the walls in each arrangement. Fans (AC Infinity, City of Industry, CA, USA) on each end of the tunnel produced a mild wind with a mean flow speed of 0.54 m s^-1^ (measured with a Velocicalc Air Velocity Meter Model 9535, TSI, Shoreview, MN, USA). The flow speed was the same above the obstacle field and within the corridors of the obstacle field (i.e., the space between the rows of obstacles); within the rows of obstacles (i.e., immediately downstream of an individual obstacle) the flow speed dropped to 0.36 m s^-1^ (S1 File).

Freely flying honeybees *Apis mellifera* (n = 58) were collected on the campus of the University of California, Davis. Single bees were flown in the tunnel with an obstacle field of one of the five possible heights, and the obstacle field height used for each bee was determined by a random number generator. Lights (26 Watts, full spectrum; Hagen, Mansfield, MA, USA) were alternately turned on and off at each end of the tunnel to motivate the bees to fly back and forth past the obstacle field (towards a light). Brightness in the tunnel was 436 ± 202 lux (mean ± SD). We filmed between 5 and 13 flights per bee (mean = 9), with approximately half the flights in still air and half the flights with wind, for a total of 548 recorded flights. We randomly assigned each bee to begin their flights with either wind or still air. Bees flew in headwinds (flying into the wind) or tailwinds (flying with the wind), depending on the direction they flew relative to the air flow on a given transit. We define the two flight directions in our tunnel as ‘up-tunnel’ and ‘down-tunnel’–bees experienced headwinds when flying in the up-tunnel direction with wind and tailwinds when flying in the down-tunnel direction with wind.

Flights were filmed with two synchronized Phantom v611 high-speed video cameras (Vision Research, Inc., Wayne, NJ, USA) sampling at 500 frames s^-1^, each positioned 30° from the vertical on opposing sides of the obstacle field and viewing down the length of the tunnel. Cameras were calibrated using a standard checkerboard calibration method and built-in MATLAB functions [38, 39]. This method captures lens distortion and projective geometry (using the intrinsic parameters), as well as the global positions and orientations of the cameras relative to the flight tunnel (via the extrinsic parameters).

### Kinematic analysis

We used a detection and tracking pipeline to automatically track the centroid of bees in each camera view as they transited the obstacle field. From each frame, we subtracted the background and found one or more candidate positions of the bee using MATLAB’s built-in blob detection functions. We associated these detections into a single trajectory over time using a Kalman filter and Munkres’ assignment algorithm [40]. We then used DLTdv6 [41] to check and manually correct the automatically tracked positions of bees. We also labeled the positions of obstacles in the field using DLTdv6. Using the camera calibration, we converted the two-dimensional locations of the objects in each view into three-dimensional coordinates of the bees and obstacles. We analyzed bees’ trajectories from when they entered to when they exited the obstacle field (Fig 1A), and we smoothed the trajectories with quintic spline curves [42]. For each flight, we used this position data to calculate the median altitude and the range of altitudes of the bee across its entire flight. We characterized route choice as above vs. within the obstacles based on bees’ median altitude relative to the height of obstacles during each trial (see below). To assess flight performance, we calculated two metrics: (1) ground speed–the median of the bee’s flight speed relative to the ground (i.e., without adding or subtracting the flow speed induced by the fans), based on its movement in the horizontal plane (lateral and fore-aft motion), and (2) lateral excursion, quantified by variation (i.e., interquartile range) in the bee’s lateral position relative to the tunnel’s longitudinal axis over the entire flight.

### Statistical analyses

Because we captured wild bees outdoors, brought them into a novel setting (a laboratory flight enclosure with a fixed obstacle field height), and then recorded multiple flights by each bee, we first tested whether bees displayed any consistent changes in flight behavior (e.g., due to learning or familiarity) over the course of the flight trials. We compared flight data (altitude, ground speed, and lateral excursion) from each bee’s first recorded flight to that of its last recorded flight using paired Student’s *t*-tests, with separate analyses performed for flights in wind and still air, because the presence of wind covaried with flight number.

#### Does obstacle field height or wind condition affect bees’ flight altitudes?

We first tested whether median altitude and altitudinal range (maximum minus minimum altitude) of bees changed with experimental conditions. For these tests, we used the R function ‘lme’ from the package ‘nlme’ [43] to create linear mixed-effects models (LMM) with terms for wind (presence vs. absence), flight direction (up- vs. down-tunnel), and obstacle field height (5 categorical levels), and we allowed for interactions between these terms. Bee identities were included as a random effect to account for multiple observations per individual. Median altitude data were squared (*x*^2^) for normality and range data were log_10_-transformed for normality.

For the median altitude comparisons, data showed significantly different variances between experimental conditions (Levene’s tests, *P* < 0.005), which violated the homogeneity of variance assumption of the LMMs. To model unequal variances across experimental conditions, we used the R function ‘varIdent’ to update the original LMM with variance structures that were weighted for each experimental group–this resulted in seven new models corresponding to all possible combinations of equal/unequal variances across levels of wind, flight direction, and obstacle field height. These models, including the original LMM, were compared by their Akaike Information Criterion (AIC) using the R function ‘AIC’. The model with the lowest AIC, which compensated for unequal variances between obstacle field heights, was used for the main analysis in place of the original LMM. The standardized residuals of the final model showed similar variances between experimental groups (Levene’s test, *P* > 0.05 for significance) and appeared to be normally distributed (checked visually using quantile-quantile plots) [44].

#### Does flight altitude or wind condition affect bees’ flight performance?

We analyzed the two flight performance metrics (ground speed, lateral excursion) relative to flight altitude and wind condition. We defined altitude (using the median for each flight) relative to the obstacle field, in three ways: altitude above the tunnel floor and in the context of each obstacle field height (Altitude_floor_ * Obstacle height), altitude relative to the top of the obstacle field (Altitude_obstacle_), and binomially, whether altitude was above or within the obstacle field (Route). These three definitions are closely correlated because (1) Altitude_obstacle_ = Altitude_floor_—Obstacle height and (2) Route = “above the obstacles” if Altitude_obstacle_ > 0 and “within the obstacles” if Altitude_obstacle_ ≤ 0. To consider each of these definitions, we analyzed flight performance with three candidate LMM models that included an altitude term based on each of these definitions. All models included terms for wind and direction, and bee identity was included as a random effect. Interactions were allowed between the altitude, wind, and direction terms. Ground speed data were log_10_-transformed for normality, and lateral excursion data were cubic-root-transformed for normality. Assumptions of homogeneity of variances were checked with Levene’s test (*P* > 0.05 for significance) and assumptions of normality were checked visually using quantile-quantile plots [44]. For each flight performance metric, the candidate models were compared by AIC and the model with the lowest AIC was used to further analyze model terms. When applicable, multiple comparisons of model terms were done with Tukey Honest Significant Difference tests using the R package ‘lsmeans’ [45]. All statistical analyses were completed with R Statistical Software [46], using a critical *P-*value of 0.05 to determine statistical significance.

## Results

Our analysis showed that the flight behavior of bees did not change in a consistent way over the course of the flight trials, in either still air or in wind (Student’s *t*-tests, *P* > 0.05; see S1 File). Thus, we were able to treat the flight trials recorded from each individual as independent from one another.

### Does obstacle field height or wind condition affect bees’ flight altitudes?

**Fig 2:**
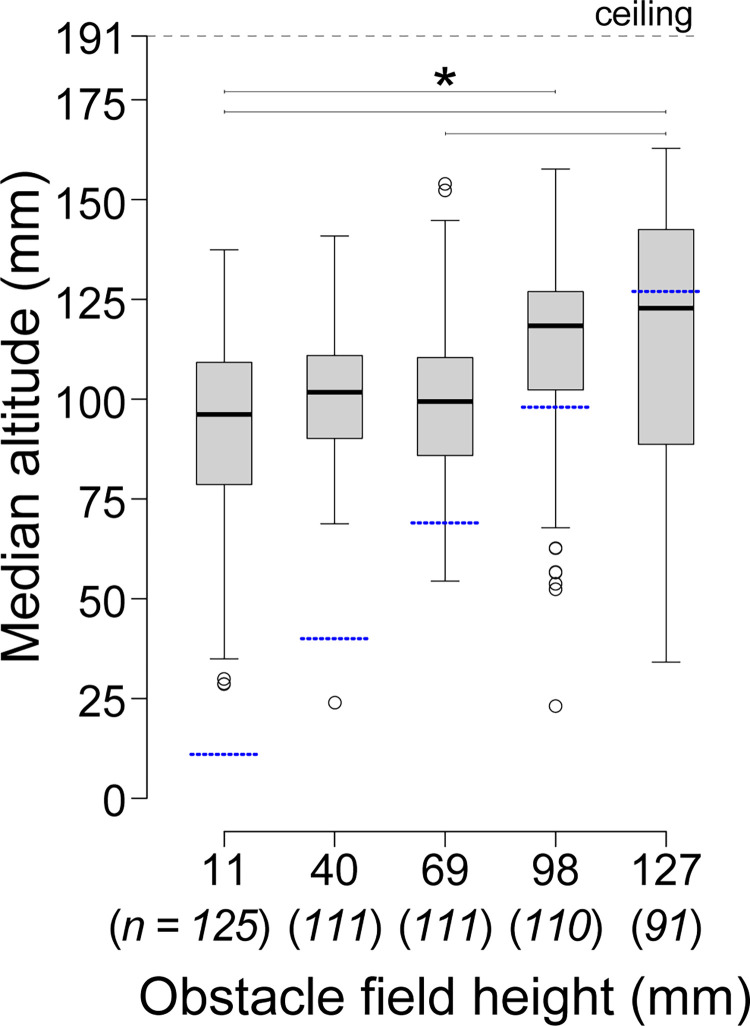
Median altitude of bees transiting obstacle fields. Dashed blue lines in indicate the obstacle field height relative to the flight altitude. Lines with asterisks indicate statistically different groups based on a linear mixed-effect model with Tukey post hoc comparisons (* *P* < 0.05). Italicized numbers below the x-axis labels show the sample size (number of flights) for each obstacle field height.

Our data revealed that trial conditions had only a minor effect on bees’ altitudes (Table 1). Median altitude depended on obstacle field height (F_(4,53)_ = 5.795, *P* < 0.005), with significant differences between the control (shortest) and two highest obstacle heights: median altitude increased by 22.2 mm (23%) from the 11-mm obstacle fields to the 98-mm obstacle fields (df = 53, t-ratio = -3.634, *P* = 0.006), and increased 26.6 mm (28%) from the 11-mm obstacle fields to the 127-mm obstacle fields (df = 53, t-ratio = -3.881, *P* < 0.005) (Fig 2). Flight direction had a moderate effect on median altitude (F_(1,475)_ = 4.447, *P* = 0.036), but there were no significant pairwise differences in median altitude between the two flight directions (df = 475, t-ratio = -1.018, *P* = 0.309) and no effect of wind on median altitude (F_(1,475)_ = 0.760, *P* = 0.384).

**Table 1:** Major behavioral responses of bees transiting obstacle fields that vary in height and wind condition.

Flight variable	Treatment response
Altitude	• Lower in 11-mm field vs. 98- and 127-mm field
Ground speed	• Faster above vs. within obstacle fields • Faster in tailwinds vs. headwinds and still air (down-tunnel)
Lateral excursion	• Larger above vs. within obstacle fields • Larger in wind vs. still air

The treatment responses describe statistically significant (*P* < 0.05) patterns in bee flight behavior.

Bees maintained a relatively narrow range of altitudes as they transited the obstacle field (median range = 20.9 mm), and altitudinal range did not change with wind (F_(1,475)_ = 2.213, *P* = 0.138), flight direction (F_(1,475)_ = 0.356, *P* = 0.551), or obstacle field height (F_(4,53)_ = 2.535, *P* = 0.051). Overall, bees displayed similar altitudinal ranges across all conditions, and although there were a few statistically significant differences in median altitude, absolute differences in altitude were minor; for example, median flight altitude increased by only 26.6 mm between the shortest and tallest canopies, despite a 116-mm increase in obstacle field height (Fig 2).

### Does flight altitude or wind condition affect bees’ flight performance?

**Fig 3:**
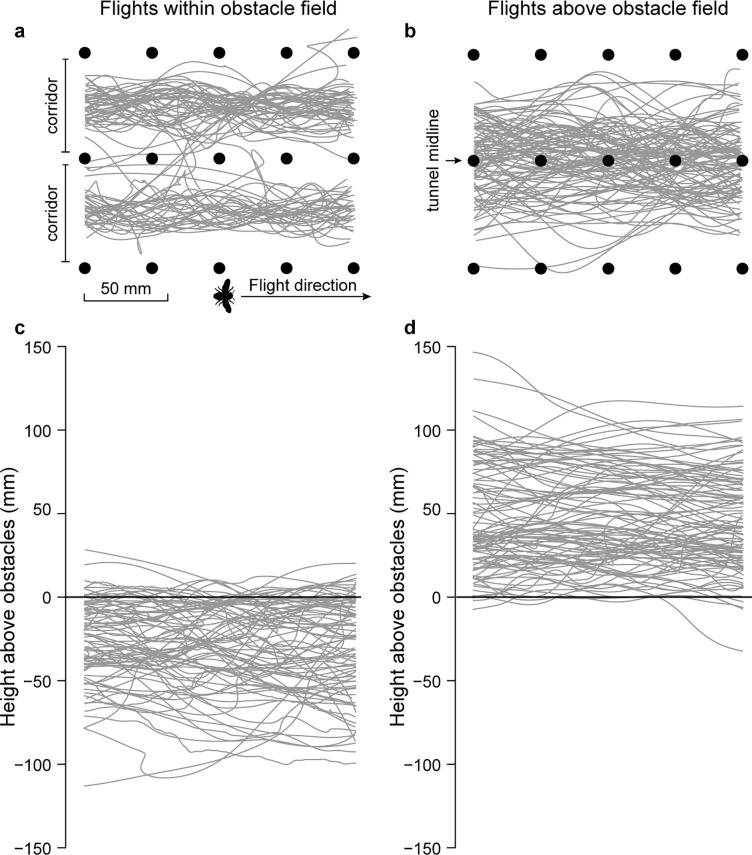
Digitized flight trajectories for bees flying within (a, c) and above (b, d) the obstacle field. Panels (a) and (b) show trajectories from a top-view, illustrating the longitudinal and lateral extent of the flight paths. Panels (c) and (d) show trajectories from a side-view, illustrating the longitudinal and vertical extent (relative to the top of the obstacle field) of the flight paths. All 82 flights within the obstacle field are shown, but for clarity, traces of only 100 of the 466 flights above the obstacle field are shown.

**Fig 4:**
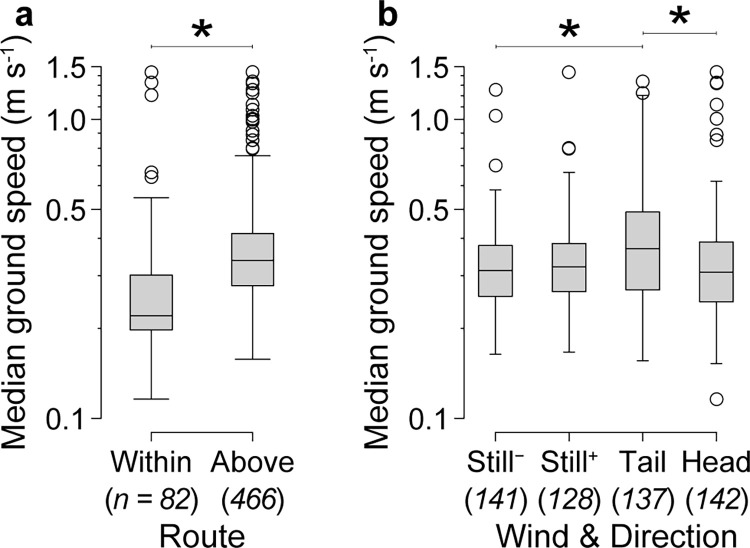
Effects of route and wind on ground speeds of bees transiting the obstacle field. (a) Median ground speeds were significantly higher above versus within the obstacle fields. (b) Median ground speeds were significantly higher in tailwinds than in headwinds and still air (down-tunnel direction only). “Still^-^” indicates down-tunnel flights in still air and “Still^+^” indicates up-tunnel flights in still air. Lines with asterisks indicate statistically different groups based on a linear mixed-effect model with Tukey post hoc comparisons (* *P* < 0.05). Italicized numbers below the x-axes labels show the sample size (number of flights) for each group.

**Fig 5:**
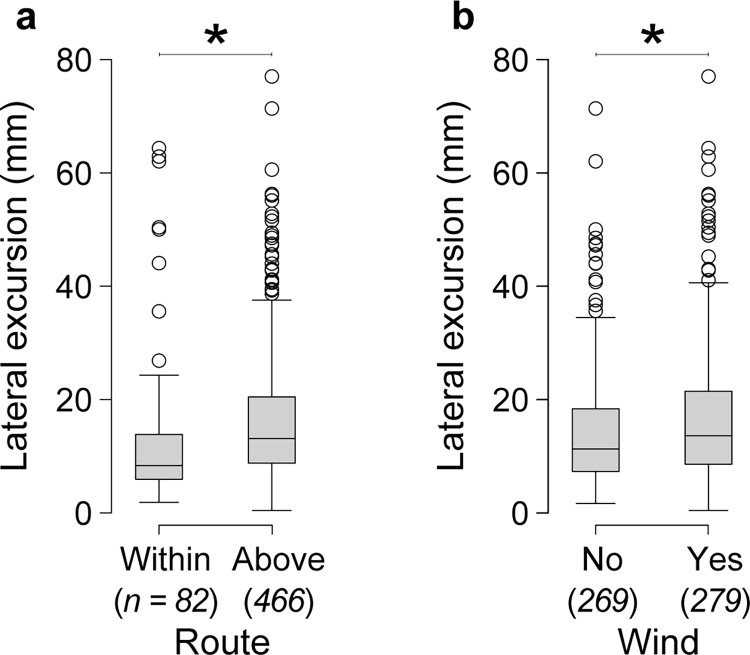
Effects of route and wind on lateral excursions of bees transiting the obstacle fields. Lateral excursions were (a) larger above versus within the obstacle fields and (b) larger in wind versus still air. Lines with asterisks indicate statistically different groups based on a linear mixed-effect model (* *P* < 0.05). Italicized numbers below the x-axes labels show the sample size (number of flights) for each group.

We tested whether ground speed and lateral excursions were affected by flight altitude, wind, and flight direction, using three definitions of altitude: median altitude relative to the top of the obstacle field, median altitude relative to the tunnel floor (in addition to a term for obstacle field height), and categorically, whether median altitude was above or within the obstacle field. We found that variation in both ground speed and lateral excursions was best explained by a model that used the categorical definition of altitude (above versus within the obstacle field; Fig 3). Ground speed was 40% faster when bees flew above rather than within the obstacle field (F_(1,483)_ = 19.542, *P* < 0.005), and these speeds were relatively stable in each route–the change in speed from the entrance to the exit of the obstacle field was -0.01 ± 0.19 m s^-1^ above the obstacle field and 0.03 ± 0.26 m s^-1^ within the obstacle field. Ground speed was also 19% faster when bees were flying with tailwinds versus headwinds (df = 483, *t*-ratio = 3.590, *P* < 0.005) and 23% faster in tailwinds than in still air during down-tunnel flights (df = 483, *t*-ratio = 4.722, *P* < 0.005) (Fig 4). As with the two route choices, the change in ground speed from the entrance to the exit of the obstacle field was minimal in each flight direction and wind conditions (mean change in speed for each group ranged from -0.03 to 0.01 m s^-1^). Lateral excursions were 36% larger when bees flew above rather than within the obstacle field (F_(1,483)_ = 7.096, *P* = 0.008) and 19% larger in wind (either headwinds or tailwinds) than in still air (F_(1,483)_ = 12.888, *P* < 0.005) (Fig 5). Lateral excursions were not affected by flight direction (F_(1,483)_ = 1.685, *P* = 0.195).

## Discussion

### Does obstacle field height or wind condition affect bees’ flight altitudes?

Honeybee flights in the three shortest obstacle fields were concentrated around an altitude of approximately 100 mm, nearly halfway between the tunnel floor and the tunnel ceiling (Fig 2). When obstacle field height surpassed this midpoint, bees increased their altitudes by approximately 20%. These results suggest that bees chose to fly at a preferred altitude that was determined by the dimensions of the flight tunnel (which affects optic flow from the walls, floor, and ceiling) and only responded to the obstacle field when it interfered with their preferred altitude. Bees’ modest increase in median altitude with higher obstacles allowed them to fly above the obstacles in the majority of trials with the 98-mm field, but with the highest field (127-mm), bees flew within the obstacles during more than half of the trials, despite having adequate space to avoid the obstacles entirely. These results suggest that bees generally prefer to fly above obstacle fields, which is not surprising given that bees tend to fly routes that maximize the distance between their bodies and any surrounding landscape features [11, 16, 47]. We expect that in larger flight arenas, and in nature more generally, bees will fly at higher altitudes as set by the proximity of obstacles and the optic flow they provide the bees. This flight strategy can minimize the risk of collisions with obstacles. For example, although the present study did not involve a particularly challenging arrangement of obstacles, with straight flight corridors approximately 3X wider than the bees’ wing spans, 6% of the 82 flights within the obstacle fields still resulted in a collision. Thus, bees tended to adjust their altitudes higher to fly in the open space above the obstacle fields; however, they were not strongly deterred by the obstacles and did not consistently avoid flying through them, despite having the space to do so.

Flight altitude was not affected by wind or flight direction. This result was not surprising because the obstacle field did not significantly attenuate wind speed [48], and the wind speed used in the experiment (~0.54 ms^-1^) was not particularly challenging compared to winds in which honeybees are capable of flying [49]. If the obstacle fields had been arranged in a manner that did attenuate wind speeds (e.g., with staggered obstacles and/or closer obstacle spacing), bees might have chosen to fly within the obstacles more often to experience lower wind speeds; however, the benefit of lower wind speeds in tightly clustered obstacle fields might be offset by the increased cost of maneuvering around obstacles, the increased risk of collisions with obstacles, or increased turbulence caused by air moving around the obstacles [28]. More studies investigating bees’ use of vegetation and clutter as a refuge from wind are needed to fully understand how they weigh the risk of collisions and cost of maneuvering around obstacles against the altered wind conditions that the obstacles may offer.

### Does flight altitude or wind condition affect bees’ flight performance?

Variation in ground speed and lateral excursion were best explained by models that considered whether median flight altitudes were above versus within an obstacle field (Fig 3), but not necessarily how far the bees were above or below the obstacle fields or the tunnel floor. These results suggest that there is a sharp transition in bees’ flight performance based on whether they are above versus within an obstacle field, rather than a gradual transition in flight performance depending on how far they are above or below the obstacles.

Flights within the obstacle fields were characterized by reduced ground speeds and narrower lateral excursions (Figs 4 and 5). Bees likely flew more slowly within the obstacle fields as a way to balance the close proximity of the obstacles and maintain a preferred optic flow rate [12, 16, 37]. Although bees were free to fly between the two obstacle corridors, most bees remained within a single corridor (Fig 3), suggesting that the narrow lateral excursions bees displayed when flying within the obstacle field were due to the reduced flight space within a single corridor. Correspondingly, we expect that bees in larger flight arenas, such as those used in other studies [17], where there are longer distances between the bee and nearby obstacles, will fly faster to maintain a preferred optic flow rate. We also expect that bees will exhibit larger lateral excursions as provided by the available flight space. These data also suggest that the reduced ground speeds for flights within the obstacle field were not due to bees attempting slow, controlled turns around obstacles [6], as this behavior would have increased the magnitude of lateral excursions. Thus, the rapid transition in flight performance as bees flew within the obstacle field is likely due to the visual feedback and narrow flight space provided by the obstacles in the field.

Wind has a significant effect on flight performance, affecting both ground speed and lateral excursion in different ways. Ground speed was affected only by tailwinds, in which bees flew 12–19% faster than in the three other wind and flight directions (Fig 4). This suggests that it may have been difficult for bees to maintain their preferred ground speed in tailwinds, when air was flowing from back to front and pushing the bee forward. For example, with a tailwind of 0.54 m s^-1^ and a preferred ground speed of 0.32 m s^-1^ (the median ground speed in still air), bees would need to fly *backwards* relative to the wind at approximately 0.22 m s^-1^ to maintain their preferred ground speed. The similarity in ground speeds between flights in headwinds and in still air is in accordance with previous studies showing that insects, including honeybees, are able to maintain their preferred ground speed when flying in a headwind [21, 50–53]. Thus, tailwinds presented a unique flight challenge for honeybees within the context of our experimental set-up.

In contrast to ground speed, lateral excursions were affected by *both* headwinds and tailwinds, with bees displaying increased lateral excursions in these wind conditions. Because lateral excursions increased in both headwinds and tailwinds, this effect is likely related to navigation rather than force production (e.g., air speed control). Mechanical stimuli from wind can interfere with insects’ responses to visual stimuli [52], so the larger lateral excursions that bees displayed when flying in wind may have been an intentional adjustment that allowed the bees to acquire more visual information from the landscape and better compensate for the mechanical challenge of wind [22, 23]. To our knowledge, most studies on the effects of wind on bee flight have been conducted in headwinds [21, 49], so additional studies that examine how steady winds from other directions interact with visual signals to affect flight performance are needed [54].

## Conclusions

Here we present data suggesting that the route choices of bees (specifically their chosen flight altitude) can be affected by large-scale properties of the surrounding landscape. Bees only engage with clutter, such as vegetation, if it encroaches on their preferred flight altitude; however, bees do not seem to be strongly deterred by clutter and do not consistently avoid flying through it, even when they are able to do so. Bees’ choices of whether to fly within clutter or to avoid it entirely can alter their flight performance: flights within clutter are slower and bees display narrower lateral casting motions, likely because of visual feedback from nearby obstacles and an increased risk of collisions. Wind does not appear to affect bees’ route choices (at least in the context of this study), but it does alter flight performance. Bees’ ground speeds are significantly higher in tailwinds, likely due to difficulties with flight control, and bees display wider lateral casting motions in all windy conditions, possibly to gain additional visual information. Thus, even in this small-scale laboratory experiment, it is clear that the challenges imposed by physically complex environments can impact bees’ behavior and flight mechanics for myriad reasons. Identifying the nuances of these effects and their underlying mechanisms can help us understand how honeybees in large-scale natural environments respond to similar types of challenges, and how resulting changes in behavior and flight performance may affect their foraging efficiency, pollination, and mortality.

## Supporting information

S1 File Supporting information for materials and methods. This file includes details of the wind conditions within the flight tunnel, the material lining the walls of the flight tunnel, and statistical summaries of the change in flight behaviors of the course of the experiments.
(PDF)
